# Multi-Algorithm-Integrated Tertiary Lymphoid Structure Gene Signature for Immune Landscape Characterization and Prognosis in Colorectal Cancer Patients

**DOI:** 10.3390/biomedicines12112644

**Published:** 2024-11-19

**Authors:** Xianqiang Liu, Dingchang Li, Yue Zhang, Hao Liu, Peng Chen, Yingjie Zhao, Guanchao Sun, Wen Zhao, Guanglong Dong

**Affiliations:** 1 Medical School of Chinese PLA, Beijing 100853, China; dr_liuxianqiang@163.com (X.L.);; 2 Department of General Surgery, The First Medical Center, Chinese PLA General Hospital, Beijing 100853, China; 3 School of Medicine, Nankai University, Tianjin 300071, China

**Keywords:** immune cell infiltration, bioinformatics analysis, consensus clustering, immunotherapy, tumor microenvironment, machine learning

## Abstract

Purpose: Colorectal cancer (CRC) is a common malignancy with a low survival rate as well as a low response rate to immunotherapy. This study aims to develop a risk model based on tertiary lymphoid structure (TLS)-associated gene signatures to enhance predictions of prognosis and immunotherapy response. Methods: TLS-associated gene data were obtained from TCGA-CRC and GEO cohorts. A comprehensive analysis using univariate Cox regression identified TLS-associated genes with significant prognostic implications. Subsequently, multiple algorithms were employed to select the most influential genes, and a stepwise Cox regression model was constructed. The model’s predictive performance was validated using independent datasets (GSE39582, GSE17536, and GSE38832). To further investigate the immune microenvironment, immune cell infiltration in high-risk (HRG) and low-risk (LRG) groups was assessed using the CIBERSORT and ssGSEA algorithms. Additionally, we evaluated the model’s potential to predict immune checkpoint blockade therapy response using data from The Cancer Imaging Archive, the TIDE algorithm, and external immunotherapy cohorts (GSE35640, GSE78200, and PRJEB23709). Immunohistochemistry (IHC) was employed to characterize TLS presence and CCL2 gene expression. Results: A three-gene (CCL2, PDCD1, and ICOS) TLS-associated model was identified as strongly associated with prognosis and demonstrated predictive power for CRC patient outcomes and immunotherapy efficacy. Notably, patients in the low-risk group (LRG) had a higher overall survival rate as well as a higher re-response rate to immunotherapy compared to the high-risk group (HRG). Finally, IHC results confirmed significantly elevated CCL2 expression in the TLS regions. Conclusions: The multi-algorithm-integrated model demonstrated robust performance in predicting patient prognosis and immunotherapy response, offering a novel perspective for assessing immunotherapy efficacy. CCL2 may function as a TLS modulator and holds potential as a therapeutic target in CRC.

## 1. Introduction

Colorectal cancer is one of the leading causes of cancer-related deaths [1]. In 2022, nearly 151,030 new CRC incidences and nearly 52,580 CRC-related mortalities were reported in both genders. These numbers will continue to rise [2]. During initial diagnosis, liver metastasis is observed in approximately 20–25% of patients with metastatic CRC, and nearly 50% of liver metastases occur in patients undergoing resection of primary cancer [3,4,5]. Furthermore, the prospects of successful surgical intervention for patients with distant metastases are notably limited. Therefore, the patient’s prognosis in advanced CRC is poor, with only a 15% 5-year survival rate [6,7]. In CRC, tumor diameter, distant metastases (to the liver and lungs), non-random chromosomal aberrations, and mutations in genes, such as EGFR, RAS, and BRAF, affect the patient’s prognosis and response to immuno- and radio-therapies as well as other treatment strategies [8,9,10]. However, the clinicopathological features of patients with similar clinical stages could differ. This indicates that the patient’s prognosis based on conventional clinicopathological staging may not be completely accurate [11]. Therefore, there is a need to identify molecular targets and prognostic biomarkers to improve the quality of life and prognosis of CRC patients. This would aid in designing personalized therapeutic strategies for patients.

Lymphoid tissues, such as tertiary lymphoid structures (TLSs) or ectopic lymphoid organs, are very similar in structure to secondary lymphoid organs [12,13]. The development of fundamental lymphoid tissues, such as secondary lymphoid organs, begins during embryogenesis. TLSs develop only in non-lymphoid tissues with chronic inflammation, such as chronic infection, autoimmune diseases, and cancers [12,14,15,16,17]. The mature TLSs are areas enriched in B cells consisting of B cell follicles surrounded by T follicular helper cells and follicular dendritic cells (DCs) and areas enriched in T cells, along with DCs, high endothelial minor veins, and lymphatic vessels [18,19]. Studies have shown that TLS allows cytotoxic T lymphocytes (CTL) to infiltrate the tumor microenvironment (TME) [20,21]. TLS is a site for producing circulating effector immune cells; hence, it regulates tumor growth [22,23]. High TLS abundance mediates anti-tumor effects, improves the efficacy of chemotherapy or immune checkpoint blockades (ICBs), and reduces cancer recurrence, thereby improving the prognosis of patients with cancers [20,24]. The role of tertiary lymphoid tissue in the TME is unique. Hence, studies have shown that TLS-associated genes could be used to predict patient prognosis accurately [24,25,26]. Currently, the ability of TLS-associated genes to predict the patient’s prognosis and the involvement in the tumor immune microenvironment (TIME) in CRC are poorly understood. Therefore, in this study, we aimed to design a novel risk model based on TLS-related genes to accurately predict the prognosis of CRC patients and their response status to immunotherapy.

We identified three TLS-related genes using multiple algorithms. A risk model was developed based on the TCGA-CRC cohort, and risk scores were calculated to comprehensively analyze the associations among TLS, TIME, and patients’ responses to immunotherapy and chemotherapy. Additionally, immunohistochemical data verified the correlation between CCL2 and tertiary lymphoid structure formation. These findings may aid in developing novel tools and improving therapeutic outcomes.

## 2. Methods

### 2.1. Source of Patient Data

We obtained gene expression profiles and clinical data of 380 colorectal cancer patients from TCGA. In addition, we utilized the GSE39582, GSE17536, and GSE38832 datasets from the Gene Expression Omnibus (GEO) database as external validation datasets and evaluated the efficacy of immunotherapy using data from the PRJEB23709, GSE45640, and GSE78220 trial data.

### 2.2. Consensus Clustering

We employed the ConensusClusterPlus R package for segregating all samples into different clusters [27] to elucidate the TLS characteristics of patients with CRC. Next, we used heatmaps to visualize the different expressions of TLS-related genes in different clusters and the clinicopathological characteristics of the patients. We explored differences in pathways enriched in clusters of patients using genomic variation analysis (GSVA) [28]. Next, we utilized the single-sample gene set enrichment analysis (ssGSEA) algorithm for determining the infiltration of immune cells and immune checkpoint gene expression in these clusters. Further, we analyzed the difference in the OS of patients in these clusters using the ClusterSurvival R package to determine the ability of the risk model to predict the survival of patients in different clusters. Next, we conducted Kyoto Encyclopedia of Genes and Genomes (KEGG) pathway enrichment analysis to identify the associated pathways such as apoptosis, transporter proteins, cell adhesion molecules, and hematopoietic cell lineage enriched by different clusters.

### 2.3. Model Construction and Validation

**Figure 1:**
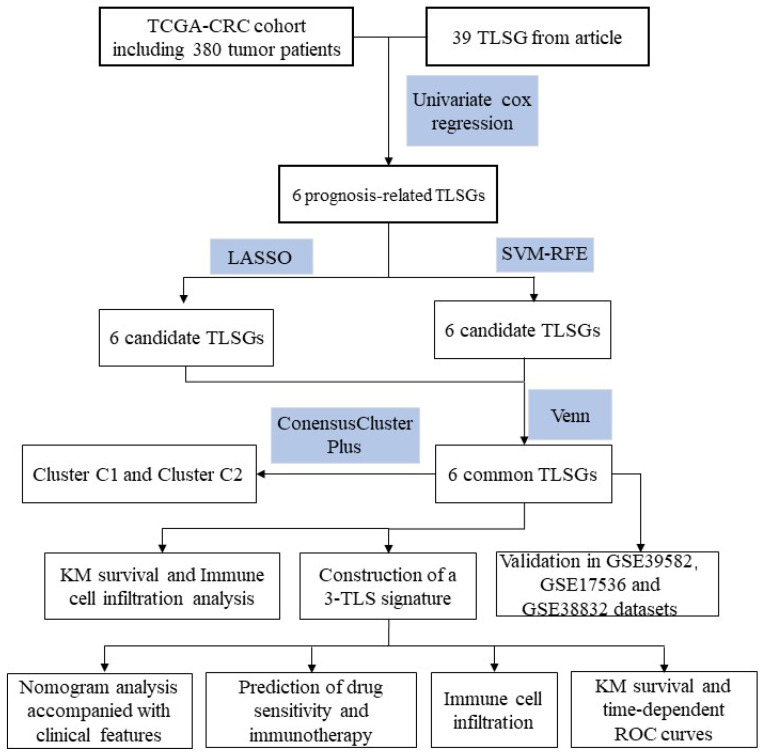
Flow chart of this study.

Figure 1 shows the flow chart of the study design. We obtained 39 TLS-associated genes from previous studies [19]. By univariate Cox regression analysis, we identified six TLS-related genes associated with prognosis. Next, we performed least absolute shrinkage and selection operator (LASSO) regression analysis to look for genes highly relevant to prognosis by tenfold cross-validation [29]. Then, we further refined the set of feature genes screened by LASSO using support vector machine (SVM)-RFE to identify the genes that contributed most to the model through a recursive process [30]. Finally, we identified and calculated the coefficients of key genes by stepwise multivariate Cox regression analysis and constructed a risk model based on the three TLS-related genes.

### 2.4. Formula of the Risk Model and Nomogram

We calculated RiskScores for all patients according to the above formula and divided them into the low-risk group (LRG) and high-risk group (HRG) based on the median. Then, we plotted the KM survival curves of patients in both groups. Further, we plotted the receiver operating characteristic curve (ROC) curves to determine the predictive ability of these TLS-associated genes. To understand the difference in the prognosis of patients in both groups, we divided all patients based on age (> and ≤65 years), gender, and clinical stages (early and advanced). We then performed survival analyses of these subgroups to determine the survival of LRG vs. HRG. In addition, we performed univariate and multivariate Cox regression analyses to determine whether risk score was an independent prognostic factor.

### 2.5. Functional Enrichment Analysis

We performed functional annotation and identified pathways enriched by TLS-associated differentially expressed genes (DEGs) using functional enrichment analysis. We performed gene ontology (GO) enrichment analysis and GSVA [28].

### 2.6. Analysis of Immune Function Genes Present in Risk Signatures

We used the Cell-type Identification by Estimating Relative Subsets of RNA Transcripts (CIBERSORT) and ssGSEA algorithm to determine the characteristics of immune cells in patients in both groups. Based on the expression pattern of 14 immune checkpoint genes described previously, we determined the expression of immune checkpoint genes in patients between HRG and LRG. Finally, we used The Cancer Immunome Atlas (TCIA) database and the Tumor Immune Dysfunction and Exclusion (TIDE) database [31] to predict the responses of patients to ICBs. PRJEB23709, GSE45640, and GSE78200 trial data were used to evaluate the effect of immunotherapy.

### 2.7. Mutation Analysis

We utilized the maftools package [32] for performing tumor mutation analysis [32] and calculating the tumor mutational burden (TMB). Furthermore, the oncoplot function was used to construct oncoplots for patients in HRG and LRH. Finally, we used Fisher’s exact test to determine differences in mutated genes in patients in LRG and HRG.

### 2.8. Tumor Immune Single Cell Hub (TISCH) Analysis

We used the TISCH database, which provides information on single-cell RNA sequencing specific for TME [33] and detailed annotation specific to cell types at the single-cell level. This would aid in analyzing particular gene expressions in different cell types, determining the differences in TME of patients, and determining the extent of the heterogeneity of CRC.

### 2.9. Estimating the Presence of TLS in Human CRC Tissue Samples

CRC tissue samples were collected at the PLA General Hospital, and hematoxylin–eosin (H&E) staining along with CD20 antibody IHC staining (GB14030, Servicebio, Wuhan, Hubei, China, 1:200) was performed. Two pathologists independently evaluated all CRC samples for the presence of TLS, identifying three TLS-positive CRC samples in total. This study was approved by the Ethics Committee of the PLA General Hospital. Subsequently, CCL2 antibody IHC (GB11199, Servicebio, Wuhan, Hubei, China, 1:500) was applied to the TLS-positive samples to detect CCL2 expression levels in formalin-fixed paraffin-embedded (FFPE) specimens. ImageJ (version 1.53k) was used for semi-quantitative analysis of the positive area percentage.

### 2.10. Statistical Analyses

The data were statistically analyzed using the “R v4.1.3” software. First, differences in OS between HRG and LRG were determined using Kaplan–Meier (KM) survival curves and log-rank tests. Then, we employed a time-dependent ROC to determine the predictive performance of the model. Finally, we used the Wilcoxon signed-rank test to compare the expression of TLS-associated genes and immune checkpoint genes, as well as the immune functions of the two groups.

## 3. Results

### 3.1. Consensus Clustering Shows the Molecular Subtypes of TLSs

**Figure 2:**
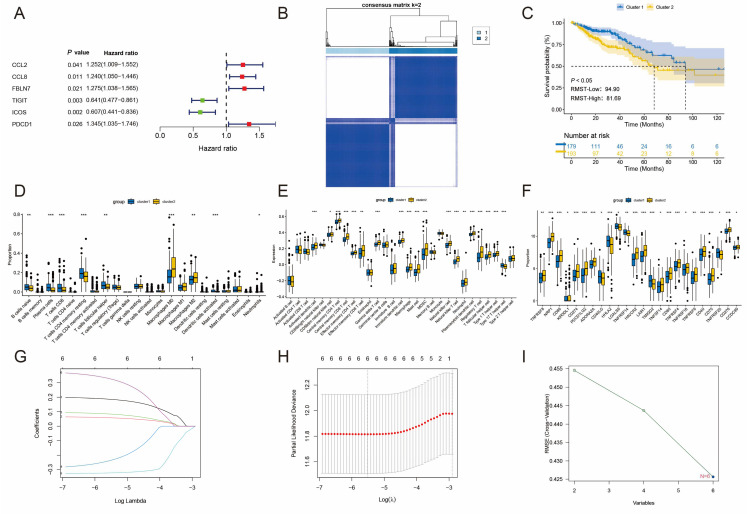
Colorectal cancer subtypes are classified by TLS-related genes. (**A**) Forest plot of TLS genes associated with prognosis. The blocks of the graph represent the HR; when the HR is greater than 1, the blocks are red, and less than 1 is green. (**B**) Heatmap of consensus matrix. (**C**) Survival analysis of C1 and C2. (**D**,**E**) Immune cell scores in C1 versus C2. (**F**) Differences in expression of immune checkpoints between C1 and C2. (**G**,**H**) Optimal number of genes identified by lasso. (**I**) SVM-RFE validates the optimal number of characterized genes. * *p* < 0.05, ** *p* < 0.01, *** *p* < 0.001.

To elucidate the underlying mechanisms behind the distinct features of TLS-associated gene expression patterns, we conducted an analysis to identify six TLS-associated genes significantly associated with the overall survival (OS) of patients with CRC using univariate Cox regression analysis (Figure 2A). These genes were subjected to unsupervised clustering, and the rationale behind the grouping was validated using cumulative distribution function (CDF) curves (Supplementary Figure S1A). Trajectory plots provided a detailed representation of the groupings (Supplementary Figure S1B). Additionally, a heatmap derived from the consensus matrix highlighted strong intra-cluster and low inter-cluster correlations (Figure 2B). Notably, the analysis indicated that patients with CRC could be categorized into two distinct subtypes, denoted as C1 and C2. Survival analysis indicated that patients classified under the C1 subtype had a more favorable prognosis than those in the C2 subtype (*p* < 0.05; Figure 2C), and patients in the C1 subtype demonstrated improved Restricted Mean Survival Time (RMST). KEGG pathway enrichment analysis identified associations with cancer-related pathways such as the TGF-beta signaling pathway, ECM receptor interaction, base excision repair, and T cell receptor signaling pathway (Supplementary Figure S1C). In addition, the infiltration of immune cells was higher in patients in C2 compared to the C1 subtype (Figure 2D,E). Finally, we explored differences in immune checkpoint gene expression between the two subtypes, revealing a significant increase in immune checkpoint gene expression in patients within the C2 subtype (Figure 2F). Consequently, these TLS-associated gene signatures hold promise as novel markers for predicting the response of patients with CRC to immunotherapy.

### 3.2. Establishment and Validation of Three TLS-Associated Gene Signatures

**Figure 3:**
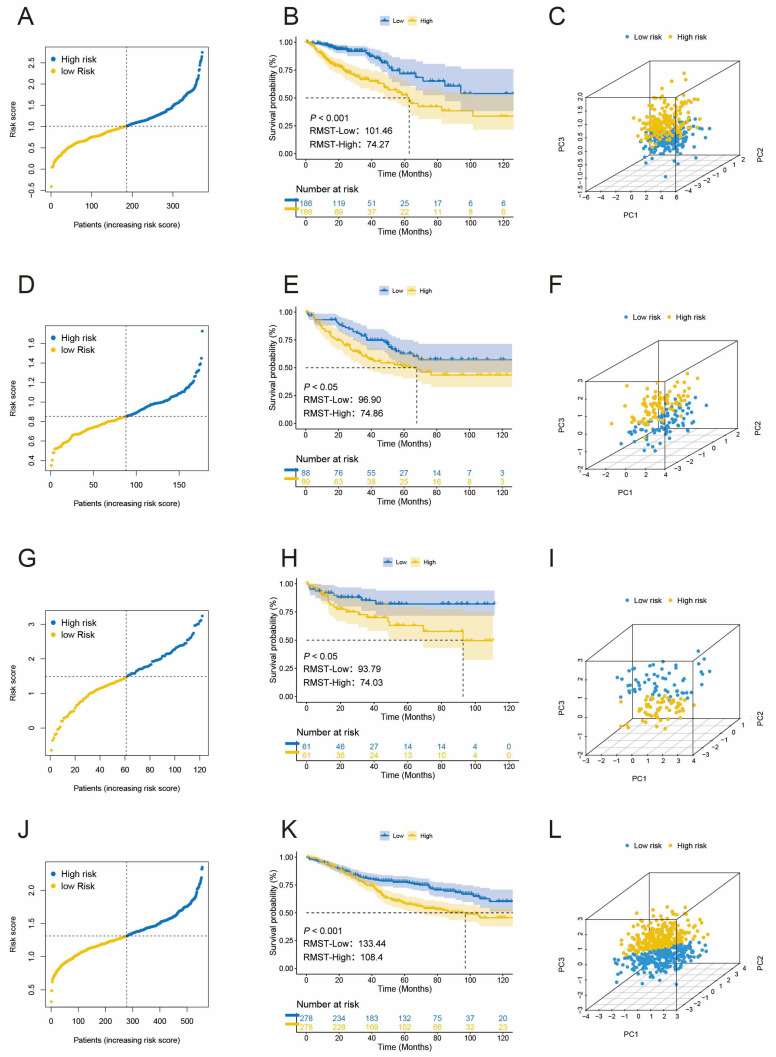
Construction and validation of three TLS-associated gene signatures. (**A**) Distribution of risk scores between LRG and HRG in the TCGA cohort. (**B**) The KM curve compares OS between LRG and HRG patients within the TCGA cohort. (**C**) PCA plot in the TCGA cohort. Risk score distribution, KM curve, and PCA plot are shown for the GSE17536 cohort (**D**–**F**), the GSE38832 cohort (**G**–**I**), and the GSE39582 cohort (**J**–**L**), respectively.

We established and validated three TLS-associated gene signatures using two algorithms: LASSO regression (Figure 2G,H) and SVM-RFE (Figure 2I). Both methods identified the same set of six genes—TIGIT, ICOS, FBLN7, CCL8, PDCD1, and CCL2—consistent with the results from univariate Cox regression. This overlap was further confirmed through Venn diagram analysis. Subsequently, we conducted a “stepwise multivariate Cox” analysis, which led to identifying three TLS-associated genes for inclusion in our RiskScore calculation. These genes are PDCD1, CCL2, and ICOS. The RiskScore calculation follows this formula: RisksScore = (0.297 × PDCD1 exp) + (0.26 × CCL2 exp) + (-0.537 × ICOS exp). We computed each patient’s RiskScore, then classified them into HRG and LRG using the median as a cutoff (Figure 3A). The prognosis of patients in LRG was favorable (*p* < 0.001; Figure 3B). To visualize the distribution of RiskScores among patients, we employed principal component analysis (PCA), revealing a marked difference and clear separation between patients in both groups (Figure 3C). Similar results were replicated in external validation datasets, where increasing RiskScores corresponded to higher patient mortality rates. PCA revealed a noticeable division between patients in each group. Kaplan–Meier survival analysis showed that in the GSE17536 (*p* < 0.05; Figure 3D–F), GSE38832 (*p* < 0.05; Figure 3G–I), and GSE39582 (*p* < 0.001; Figure 3J–L) cohorts, the prognosis for patients in LRG was better compared to HRG. Additionally, patients in the low-risk group demonstrated improved RMST.

### 3.3. Correlation Between Clinicopathological Features, Survival, and TLS-Associated Genes in Patients with CRC

**Figure 4:**
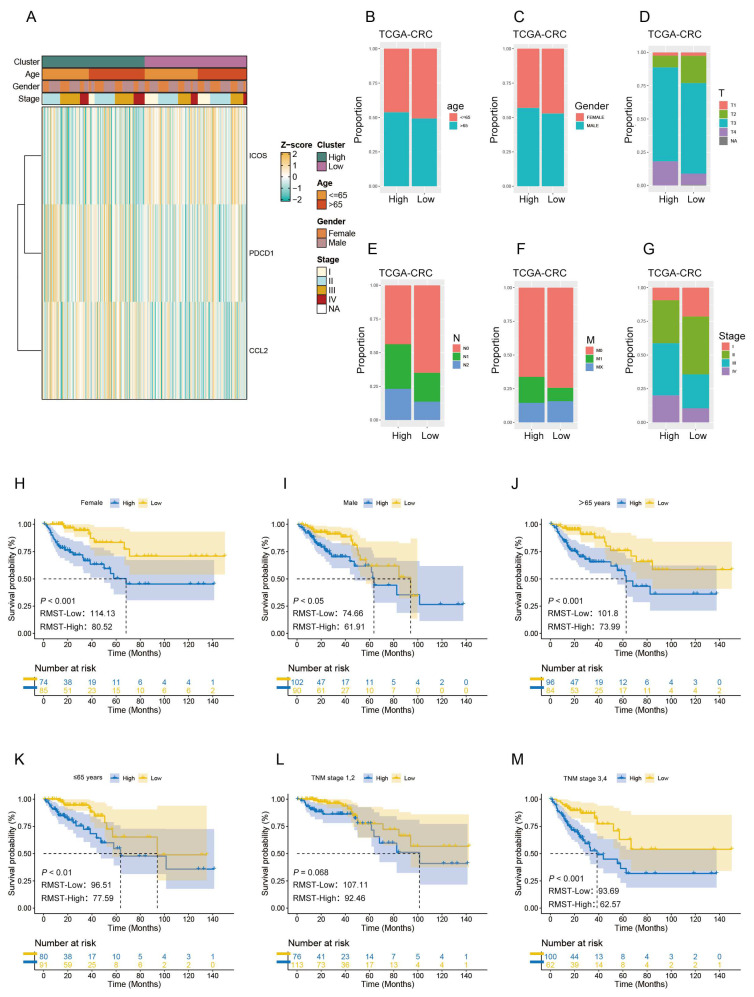
Clinical correlation and survival analysis of TLS scores in CRC patients. (**A**) Heatmap showing associations between HRG and LRG CRC patients in the TCGA dataset and clinical features. (**B**–**G**) Stacked histograms display the distribution of clinical characteristics. The TLS risk score effectively predicts poor prognosis; within subgroups defined by clinicopathologic characteristics—including (**H**,**I**) gender, (**J**,**K**) age, and (**L**,**M**) clinical stage—all HRG patients demonstrated worse outcomes.

To further demonstrate a correlation between clinicopathological features, survival, and TLS-associated genes in patients with CRC, we utilized heatmaps to analyze the correlation between clinicopathological features, RiskScores, and TLS-associated gene expressions among patients in both groups. The results highlighted significant correlations between the three TLS-associated genes and factors such as age, gender, clinical stage, and overall risk in all patients within the TCGA-CRC cohort, as illustrated in Figure 4A. Additionally, we explored the distinctions in clinicopathological features between patients in the HRG and LRG. The findings indicated a marked influence of TLS-associated genes on patients with varying clinicopathological characteristics, as demonstrated in Figure 4B–G. Furthermore, we evaluated the RMST of patients in different clinical stages, age groups, and genders within the HRG and LRG. As shown in Figure 4H–M, HRG patients exhibited lower RMST than LRG patients, except for the S12 subgroup. These results underscore the robust predictive capability of our risk model concerning patient prognosis based on their clinicopathological features.

### 3.4. Establishment of Nomograms in Combination with Clinicopathological Features

**Figure 5:**
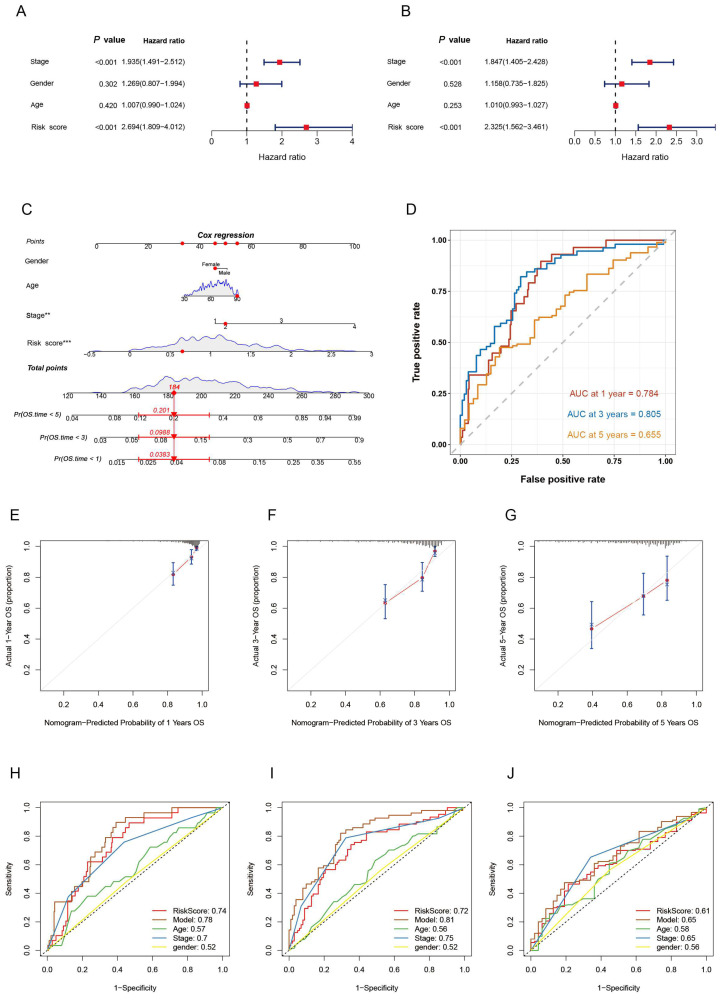
Development of nomograms integrating clinical characteristics. (**A**) COX regression analysis for univariate and (**B**) multivariate signature assessment alongside various clinical factors. The blocks of the graph represent the HR; when the HR is greater than 1, the blocks are red, and less than 1 is green. (**C**) Nomogram incorporating Riskscore, age, gender, and clinical stage. The red dots in the figure represent the scores corresponding to each of the scores for a particular patient and the relationship between the total score and the probability of survival. (**D**) multi-year ROCs. Calibration curves for the nomogram predicting survival at 1, 3, and 5 years are shown in (**E**–**G**). Time-dependent ROCs for the nomogram’s 1-year, 3-year, and 5-year survival predictions are presented in (**H**–**J**). ** *p* < 0.01, *** *p* < 0.001.

Based on the above conclusion, we have established a robust correlation between our risk model and the poor prognosis of CRC patients. To assess whether these three TLS-associated genes risk model can independently predict patient prognosis, we conducted both univariate and multivariate Cox analyses, considering overall survival (OS) and clinical characteristics. The univariate Cox and multivariate Cox analyses confirmed that the RiskScore could reliably and independently predict patient prognosis (*p* < 0.001; Figure 5A,B). Furthermore, we evaluated the practical application of the risk model in clinical settings. For this purpose, we constructed a nomogram based on patient age, sex, clinical stage, and the RiskScore to predict 1-, 3-, and 5-year OS for patients (Figure 5C). The results showed that the RiskScore was a valuable predictor of CRC patient OS (Figure 5D). To assess the accuracy of the model in predicting OS, we employed “time-dependent ROC curves.” The results revealed AUC values of 0.784, 0.805, and 0.655 for the 1-, 3-, and 5-year predictions. Additionally, calibration curves demonstrated good agreement between the predicted and observed probabilities for 1-, 3-, and 5-year OS (Figure 5E–G). Moreover, our model outperformed RiskScores, age, gender, and clinicopathological features in effectively predicting 1-, 3-, and 5-year prognoses (Figure 5H–J). Thus, our risk model demonstrated superior predictive performance compared to other features, highlighting its potential clinical utility in assessing patient outcomes.

### 3.5. Functional Enrichment Analysis of TLS

KEGG pathway enrichment analysis was conducted to identify pathways enriched by DEGs in HRG versus LRG and to assess the correlation between these pathways and the RiskScores. We selected significantly enriched terms based on |Log fold change| > 0 and adj.P.Val < 0.05 criteria. GSVA revealed 73 significantly enriched pathways (Supplementary Figure S2A). Calcium signaling pathway, Notch signaling pathway, and focal adhesion were enriched in patients in HRG. Regulating cytotoxicity mediated by the T cell receptor signaling pathway, B cell receptor signaling pathway, chemokine signaling pathway, cytokine–cytokine receptor interaction, and immune responses were enriched in patients in LRG. Taken together, the enrichment analysis was strongly correlated with immune responses; therefore, we performed a systematic analysis to determine the immune landscape of patients with CRC.

### 3.6. Risk Scores Have Predictive Value in Relation to the Tumor Microenvironment

**Figure 6:**
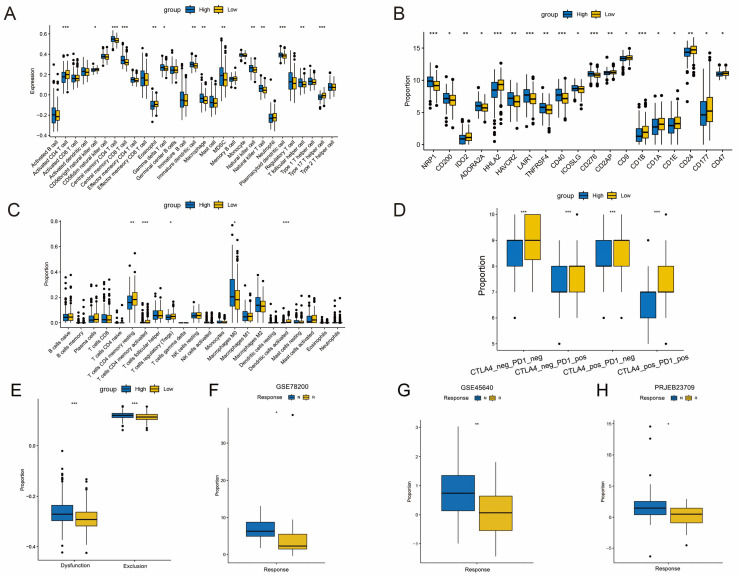
TLS risk score assessing immune cell infiltration and immunotherapy response. (**A**) ssGSEA of the 3-TLS risk model in TCGA datasets. (**B**) Differences of 19 immune checkpoints among LRG and HRG. (**C**) The CIBERSORT algorithm calculated the proportion of immune cells in the TCGA cohort (**D**). The relative distribution of immunophenoscore (IPS) between HRG or LRG. (**E**) Tumor Immune Dysfunction and Rejection (TIDE) Algorithm for Predicting Response to Immunotherapy in HRG vs. LRG Patients. The relative distribution of risk score between the Response or Non-Response group in GSE78200 (**F**), GSE45640 (**G**), and PRJEB23709 (**H**). * *p* < 0.05, ** *p* < 0.01, *** *p* < 0.001.

We employed the ssGSEA and CIBERSORT algorithms to assess immune cell infiltration in both HRG and LRG patients. Our results highlighted a significantly greater infiltration of CD8+ T cells, NK cells, dendritic cells (DCs), macrophages, and resting mast cells in LRG patients (Figure 6A,C). Considering the significance of immune checkpoint inhibitors (ICBs) in cancer immunotherapy, we also noted that LRG patients exhibited a significant upregulation in the expression of immune checkpoint genes, including HHLA2, CD2AP, CD9, IDO2, CD1A, CD1B, CD1E, CD24, CD47, and others. This suggests that patients in LRG may be more responsive to ICB therapy. To explore the impact of genetic mutations on immunotherapy, we assessed tumor mutation burden (TMB) in CRC patients (Supplementary Figure S2B,C). While no significant differences in TMB were observed between HRG and LRG, a combined survival analysis of TMB (high/low) and risk score (high/low) groups revealed that patients with low-risk + low TMB had the longest survival times (Supplementary Figure S2D).

Next, we evaluated immune characteristics and response to immunotherapy in the TCIA cohort (Figure 6D) and used the TIDE algorithm to predict ICI treatment efficacy (Figure 6E). Results showed significant differences between HRG and LRG in responses to CTLA4 and PD1 therapies (*p* < 0.001). In addition, response rates to immunotherapy were notably higher in LRG across external cohorts, including the PRJEB23709 trial (HRG: 32% vs. LRG: 60%), the GSE35640 trial (HRG: 25% vs. LRG: 53.6%), and the GSE78200 trial (HRG: 35.7% vs. LRG: 71.4%). Moreover, across all three cohorts, risk scores for CR/PR were significantly lower than those for PD/CD (Figure 6F–H), thereby indicating that patients in LRG could benefit more from immunotherapy.

### 3.7. Correlation Between Three TLS-Associated Genes and TME

**Figure 7:**
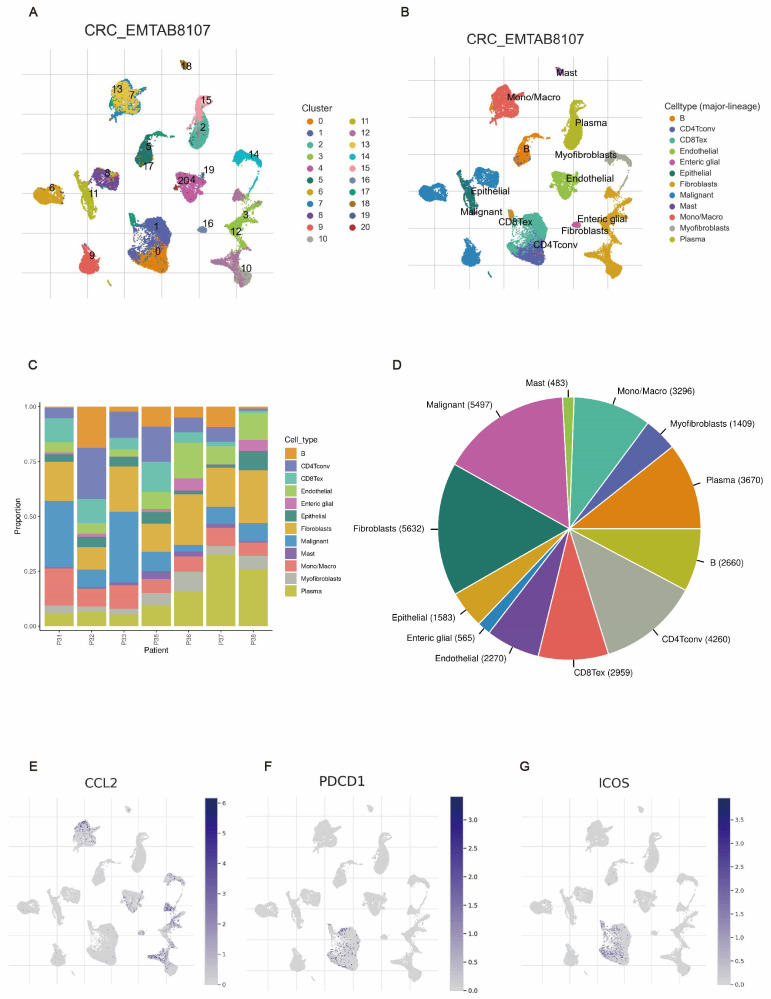
Association between three TLS genes and the tumor microenvironment. (**A**–**D**) Annotations of all cell types in the CRC_EMTAB8107 dataset. Proportions and expression levels of (**E**) CCL2, (**F**) PDCD1, and (**G**) ICOS.

We determined the expression of TLS-related genes in different cellular subpopulations using the single-cell dataset CRC_EMTAB8107 from TISCH. CRC_EMTAB8107 contained 20 cell populations and 12 immune cell types (Figure 7A,B). Figure 7C,D show the distribution and abundance of all cell types. CCL2 was expressed by all immune cells, specifically enteric glial cells, mono/macro, endothelial cells, and fibroblasts. Furthermore, PDCD1 and ICOS were mainly expressed by CD4 and CD8 T cells (Figure 7E–G).

### 3.8. Experimental Validation

**Figure 8:**
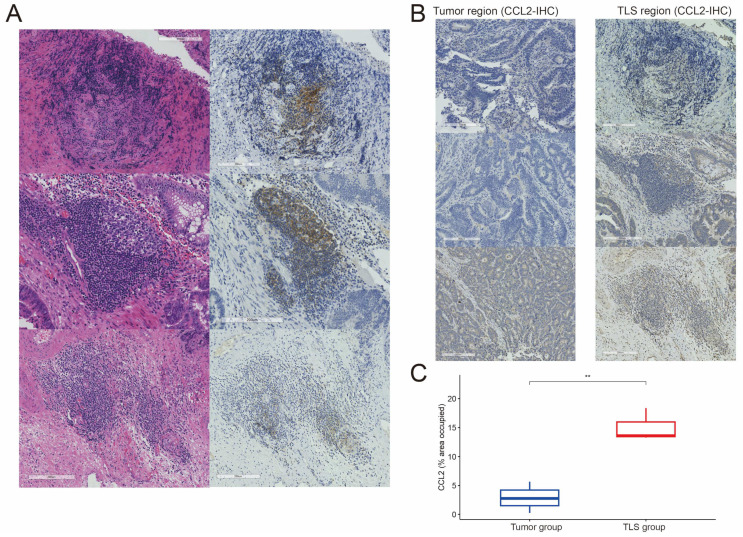
IHC and H&E staining characterized the presence of TLS and CCL2 expression in CRC and specifically within TLS regions. (**A**) H&E and CD20 IHC staining identified areas containing TLS. **(B**) CCL2 expression in both TLS regions and adjacent tumor tissue. (**C**) Differences in the proportion of CCL2-positive areas between TLS regions and tumor tissue, with ** *p* < 0.01.

IHC was used to assess CCL2 expression in both TLS regions and adjacent tumor areas. First, samples containing tertiary lymphoid structures were identified using H&E and CD20 IHC staining (Figure 8A). To further evaluate CCL2 expression within TLS regions and surrounding tumor tissue, we performed IHC on TLS+ CRC tissues (Figure 8B). The results demonstrated a significantly higher proportion of CCL2-positive areas in TLS regions compared to the adjacent tumor tissue (Figure 8C). These findings suggest that CCL2 may act as a positive regulator of TLS, potentially playing a key role in tumor metastasis and immune function.

## 4. Discussion

CRC is among the most common gastrointestinal cancers. Patients with CRC undergo surgical resection as the first-line therapy [34]. Surgical resection is more effective at the early stages; however, the prognosis of patients with advanced CRC who underwent surgical resection is poor, with a 15% 5-year survival rate [6,7]. Therefore, diagnosis of patients at an early stage and individualized therapies are critical for improving the patient’s prognosis. Currently, clinical staging and histopathology are used for diagnosis and predicting the prognosis of patients with CRC. However, due to heterogeneity and developmental trends of CRC, these indicators may not be sufficient for accurate diagnosis, prognosis, and treatment response in patients with CRC. The molecular marker demonstrates better performance in predicting the patient’s prognosis. Recent studies have demonstrated the prognostic efficacy of TLS expression in patients with cancer. A study has shown lymphocyte homing in TLS at tumor sites but not in normal tissues in a CRC mouse model [20]. Further, mature DCs in TLS directly attract CTL to infiltrate the tumor as a local immune response in lung cancer [21]. Thus, TLS is an important site for initiating and maintaining the local immune response. Based on the significance of tertiary lymphoid tissue in CRC, we obtained gene expression data on patients from the TCGA-CRC cohort to identify prognostic genes and constructed a TLS-associated gene-based risk model. We combined 39 TLS-associated gene expression patterns of patients from TCGA-CRC. Next, six TLS-associated genes, including CCL2, TIGIT, FBLN7, CCL8, PDCD1, ICOS, and CCL20, related to prognosis were identified. Further, we constructed a risk model using algorithms such as “LASSO regression”, “SVM-RFE”, and “stepwise multivariate Cox regression”. These TLS-associated genes could independently predict the patient’s prognosis. Next, we divided the patients into LRG and HRG groups based on the median RiskScore. The results showed a significant difference in prognosis between the two groups. The results were also validated in three external (GSE39582, GSE38832, and GSE17536) independent cohorts from GEO. The ROC and calibration curves demonstrated that the predictive performance of our model was excellent. Next, we constructed a nomogram based on clinicopathological features and risk scores to determine the clinical application of our model in predicting patient prognosis. Our results showed that the predictive performance of our model was better compared to the clinicopathological features of patients, which may provide guidelines for treatment decision-making.

ICB therapy, such as using PD-1/PD-L1 blockade, is the second-line treatment and has shown significant benefits in patients with unresectable and metastatic CRC. However, the rate of patient response to ICB therapy is low. Therefore, we determined if the RiskScores could predict the patient’s response to ICB therapy and aid in designing therapeutic strategies. Our results suggest that LRG patients could benefit more from immunotherapy through TIDE and TCIA analysis. We further validated this in three external real-world immunotherapy cohorts (PRJEB23709, GSE45640, and GSE78200). We found that the immunotherapy-responsive group had lower RiskScores than the non-responsive group, further confirming the validity of the RiskScores.

We performed an enrichment analysis to show a comprehensive landscape of TLS-associated gene functions. The results demonstrated that TLS-associated genes were enriched in several cancer and immune-related pathways, such as the mTOR signaling pathway, ECM receptor interaction, and Notch signaling pathway. TIIC is an important TME component. The composition and distribution of TIIC are critically involved in the occurrence and progression of cancer. Studies have shown that TME promotes inflammation and aids tumor cells in escaping immune surveillance [35,36]. Thus, a comprehensive understanding of immune cell infiltration in TME would aid in understanding the mechanism and designing immunotherapy strategies to improve clinical outcomes. Therefore, we evaluated immune cell infiltration in HRG and LRG patients. Further, CIBERSORT and ssGSEA analyses showed that central memory CD4 T cells, central memory CD8 T cells, gamma delta T cells, immature dendritic cells, macrophages, natural killer cells, natural killer T cells, plasmacytoid dendritic cells, T follicular helper cells, and macrophages (M0) were higher in HRG patients. In contrast, LRG patients were more associated with the accumulation of active dendritic cells, active CD4T cells, CD56 bright natural kill cells, eosinophil, MDSCs, and type 17 T helper cells. There is growing evidence that the presence of systemic inflammation is associated with poorer cancer-specific survival in patients with a variety of solid tumors, including colorectal cancer [37,38,39]. The interaction between the tumor and the host immune system stimulates tumor cell proliferation and metastasis, triggering the host inflammatory cascade and further deteriorating the patient’s overall condition [40]. In addition, systemic inflammation leads to further progression and metastasis of colorectal cancer and is also an independent indicator of long-term poor oncologic outcomes in terms of shorter disease-free time and overall survival [41,42]. In the immune infiltration analysis of the high-risk group, the content of most immune cells was higher than that of the low-risk group. This may explain the immune dysregulation and reduced survival of patients in the high-risk group. Studies have shown that macrophage M0 promotes cancer invasion [43]. Macrophages are immune cells that infiltrate the tumor microenvironment and play a crucial role in the immune response to cancer. Tumor-associated macrophages (TAMs) are a subtype of macrophages present in the tumor microenvironment and have been shown to promote tumor growth and metastasis [44]. M0 macrophages are a subtype of TAMs that have not been polarized to the M1 or M2 phenotypes. A study [45] found that M0 macrophages were associated with poor prognosis in liver cancer patients. Similar to our results, M0 macrophage content was higher in the HRG than in the LRG, which may contribute to the lower survival rate in the HRG. Other studies have also shown that γδ T cells stimulate interleukin-17A (IL-17A) production, which stimulates tumor cell proliferation, induces angiogenesis, and promotes inflammation [46,47]. High expression of γδ T cells in high-risk groups is also a risk factor for poor prognosis.

Based on stepwise Cox regression analysis, we identified three TLS-related genes, with the role of CCL2 in TLS formation warranting further exploration. A study has shown that chemokines alter immune response by regulating DC lymphocyte trafficking and immune homeostasis as well as surveillance [48]. The CCR2 ligand, CCL2, is secreted by cancer cells via the autocrine/paracrine pathway. CCL2 binds and activates CCR2 to attract monocytes, basophils, and macrophages to infiltrate TME, thereby promoting cancer cell invasion as well as metastasis [49,50,51,52]. Thus, blocking the CCL2-CCR2 pathway could effectively reduce the infiltration of monocytes and macrophages in the mesenchyme and inhibit tumor growth [53,54]. Additionally, this study used IHC to detect CCL2 expression within TLS regions and surrounding tumor tissue, revealing a higher proportion of CCL2 positivity in the TLS regions, suggesting a possible association between CCL2 and TLS formation.

Our research establishes a TLS gene-associated prognostic and immunotherapy evaluation model, highlighting a potential link between CCL2 and TLS formation. This model offers an innovative perspective for future research aimed at inducing TLS and holds promise for supporting personalized treatment decisions; based on these signs, further risk stratification tools for immunotherapy can be developed to assist clinicians in selecting the most suitable treatment plans and predicting patient prognosis.

### Limitation

First, as a retrospective study, there may be potential biases within the patient cohort, which could impact the reliability of the observed associations. Therefore, prospective studies are needed to validate these findings. Additionally, due to potential differences in the clinical and demographic characteristics of patients, the generalizability of our results may be limited. Future research should aim to control these confounding factors to enhance the applicability and robustness of the findings.

## Data Availability

The datasets utilized and analyzed in this study are accessible through GEO (https://www.ncbi.nlm.nih.gov/geo/; accessed on 20 May 2024) and TCGA (https://portal.gdc.cancer.gov/; accessed on 20 May 2024). For additional information, please feel free to contact the corresponding authors.

## Supplementary Materials

The following supporting information can be downloaded at: https://www.mdpi.com/article/10.3390/biomedicines12112644/s1, Figure S1: Consensus clustering identified the molecular subtypes of TLSs; Figure S2: TLS risk score predicts TME and immune cell infiltration.
