# Supplementary figures and images for: Multi-Algorithm-Integrated Tertiary Lymphoid Structure Gene Signature for Immune Landscape Characterization and Prognosis in Colorectal Cancer Patients
Source: Biomedicines. 2024 Nov 19;12(11):2644. doi: 10.3390/biomedicines12112644 (PMC11592260; doi:10.3390/biomedicines12112644)

Supplementary material

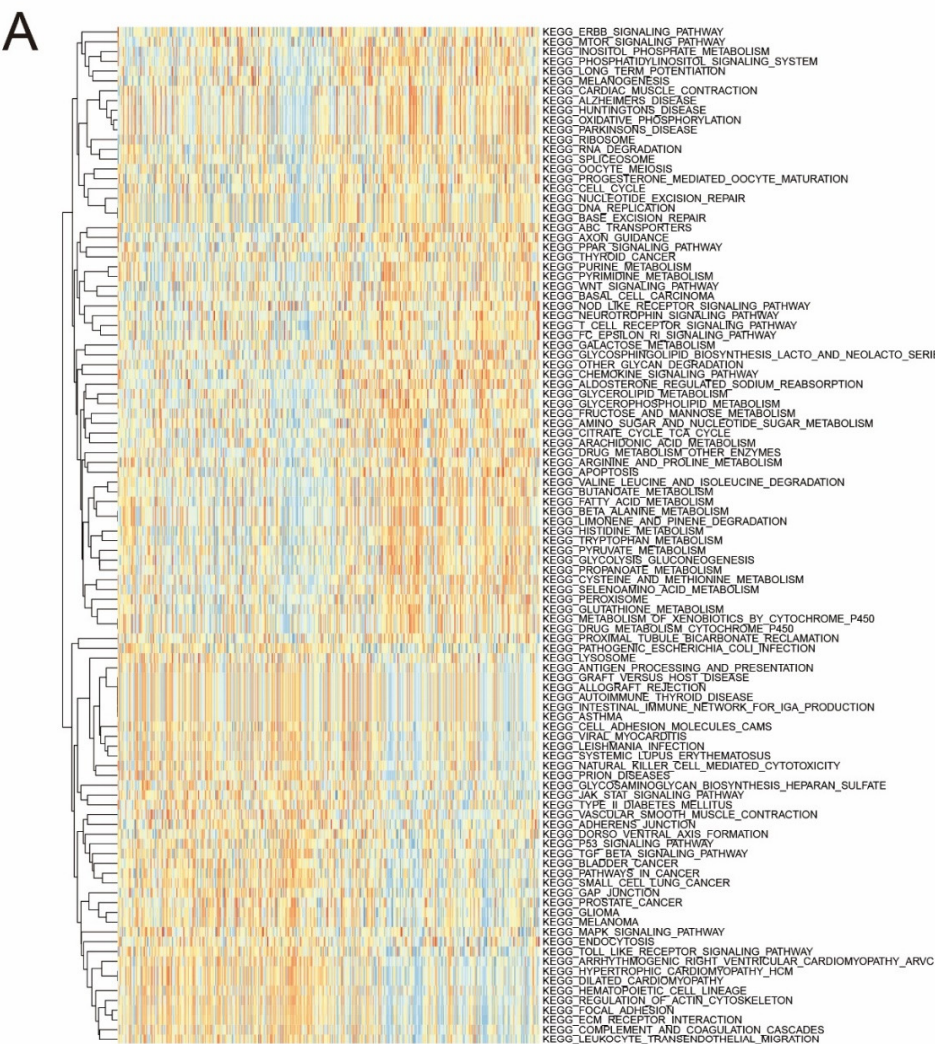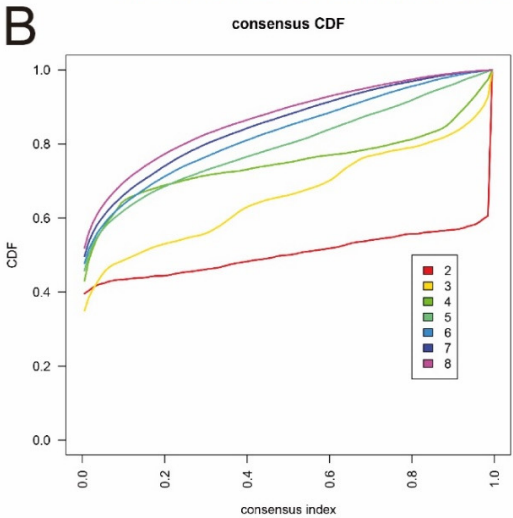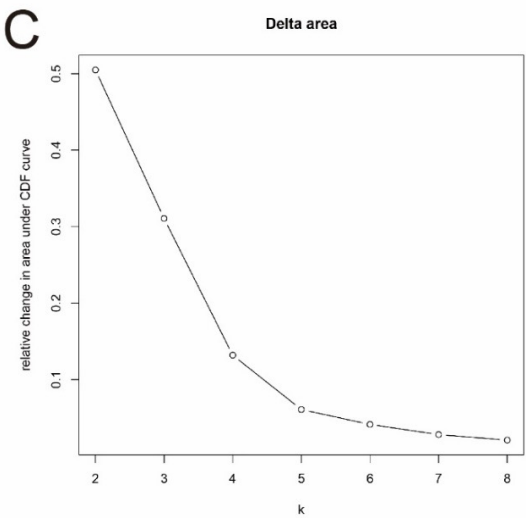

Supplement: Supplementary file 1 [file biomedicines-12-02644-s001.zip › biomedicines-3255995-supplementary.pdf]
